# Pirfenidone Improves Familial Idiopathic Pulmonary Fibrosis without Affecting Serum Periostin Levels

**DOI:** 10.3390/medicina55050161

**Published:** 2019-05-17

**Authors:** Yasuhiko Koga, Yoshimasa Hachisu, Hiroaki Tsurumaki, Masakiyo Yatomi, Kyoichi Kaira, Shoichiro Ohta, Junya Ono, Kenji Izuhara, Kunio Dobashi, Takeshi Hisada

**Affiliations:** 1 Department of Allergy and Respiratory Medicine, Gunma University Graduate School of Medicine, 3-39-15 sho-wa machi Maebashi, Gunma 371-8511, Japan; yhachisu2002@yahoo.co.jp (Y.H.); m12702056@gunma-u.ac.jp (H.T.); m09702007@gunma-u.ac.jp (M.Y.); 2 Department of Respiratory Medicine, Comprehensive Cancer Center, International Medical Center, Saitama Medical University, Saitama 350-0495, Japan; kkaira1970@yahoo.co.jp; 3 Division of Medical Biochemistry, Department of Biomolecular Sciences, Saga Medical School, 5-1-1, Nabeshima, Saga 849-8501, Japan; sho.ohta@iuhw.ac.jp (S.O.); kizuhara@cc.saga-u.ac.jp (K.I.); 4 Shino-Test Corporation, 2-29-14 Oonodai Minami-ku, Sagamihara, Kanagawa 252-0331, Japan; junya.ono@shino-test.co.jp; 5 Jobu Hospital for Respiratory Diseases, 586-1 Taguchi-machi, Maebashi, Gunma 371-0048, Japan; dobashik@gunma-u.ac.jp; 6 Gunma University Graduate School of Health Sciences, 3-39-22 sho-wa machi Maebashi, Gunma 371-8514, Japan; hisadat@gunma-u.ac.jp

**Keywords:** familial, idiopathic interstitial pneumonia, pirfenidone, idiopathic pulmonary fibrosis, periostin

## Abstract

*Background:* Antifibrotic agents have been approved for the treatment of idiopathic pulmonary fibrosis (IPF). However, the efficacy of these drugs in the treatment of familial IPF (FIPF) has not been previously reported. *Case presentation:* We report the case of a 77-year-old man with FIPF, successfully treated with pirfenidone. His uncle died due to IPF, and his niece was diagnosed with the disease. He had worsening dyspnea two months prior to admission to our hospital. Upon admission, he had desaturation when exercising and broad interstitial pneumonia. Administration of pirfenidone improved his dyspnea, desaturation, and the reticular shadow on his chest radiograph. Increased fibrotic marker levels KL-6 and SP-D were also normalized in six months; treatment had no effect on his serum periostin level. Pirfenidone has been effective for over two years. *Conclusion:* Antifibrotic agents such as pirfenidone may be useful for the management of FIPF, as well as cases of sporadic IPF.

## 1. Introduction

It is known that familial idiopathic pulmonary fibrosis (FIPF) makes up 0.5% to 2.2% of idiopathic pulmonary fibrosis (IPF) cases [1], and does not always result in the honeycomb lungs typical of sporadic IPF cases [2]. Genetic differentiation on *MUC5B* or telomerase genes has been reported [3,4]. However, effective treatment for FIPF has not been established. Here, we report a case of IPF with familial history of the disease having a good response to pirfenidone for more than two years. Fibrotic markers sialylated carbohydrate antigen KL-6 (KL-6), surfactant protein-D (SP-D), and periostin followed different time courses after pirfenidone. This is the first case report describing effective treatment for FIPF using pirfenidone and showing a time course of fibrotic markers and periostin.

## 2. Case Presentation

**Figure 1:**
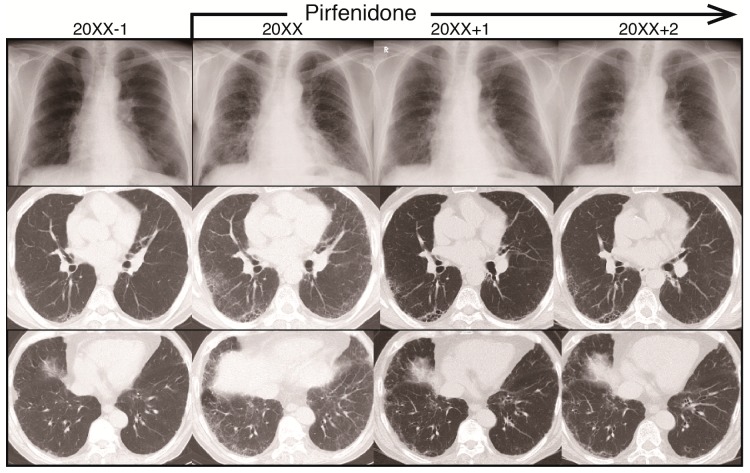
Radiograph images during the clinical course of idiopathic pulmonary fibrosis (IPF) for one year before the two years after initiation of pirfenidone treatment. Upper panel: reticular shadow on chest radiograph. Middle and lower panels: computed tomography (CT) images of influence of pirfenidone on IPF over a two-year period.

A 77-year-old man with no history of smoking was admitted to our hospital due to worsening dry cough and dyspnea on exertion over the previous two months. The previous year, he was tentatively diagnosed with asymptomatic idiopathic interstitial pneumonia (IIP) at another hospital. Reticular infiltrates on computed tomography (CT) examination, performed at the time of his initial admission to our hospital, revealed the progression of IPF when compared with CT images obtained one year previously (Figure 1).

**Figure 2:**
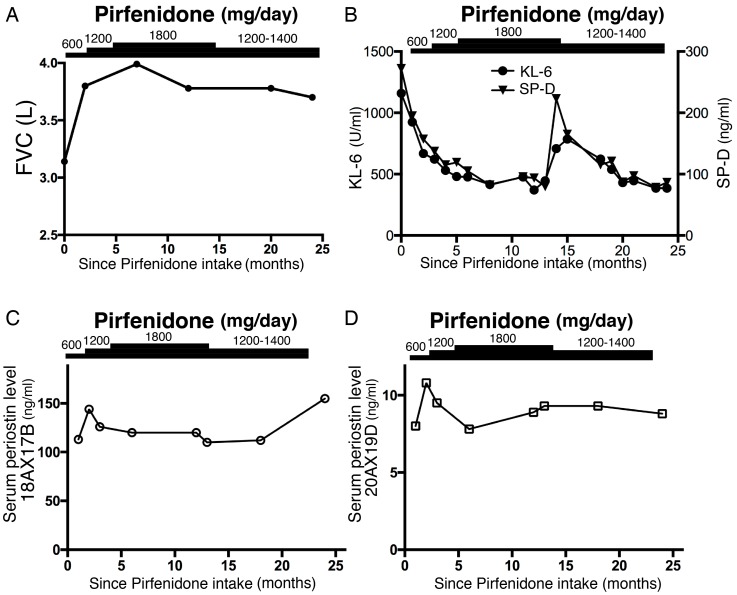
Time course of (**A**) forced vital capacity (FVC), (**B**) serum fibrotic marker KL-6 and SP-D levels, and (**C**,**D**) serum periostin levels after administration of pirfenidone. After pirfenidone treatment, (**A**) FVC was improved, and (**B**) KL-6 and SP-D levels were normalized. Periostin levels were not clearly elevated on initial admission and were not affected by pirfenidone (**C**,**D**). Antibodies 18A X 17B (<95 ng/mL) and 20A X 19D (<13.4 ng/mL) recognize total (inflammatory and fibrotic) and monomeric (fibrotic) periostin, respectively [6].

Laboratory findings did not reveal any collagen disorders associated with interstitial pneumonia (IP). These investigations, however, did reveal elevated levels of fibrotic markers KL-6 (1448 U/mL), SP-A (66.4 ng/mL), and SP-D (353 ng/mL). The patient also had a familial history of IPF: his uncle had died from it and his niece had the disease. We diagnosed this case as probable usual interstitial pneumonia pattern with bronchiectasis in the lower lung field via the multidisciplinary-discussion approach, and prescribed a low dose of pirfenidone (600 mg/day) for a month and 1200 mg/day for the following month, after which his symptoms of dry cough and dyspnea during exercising improved. Although the history of cough occurrence/persistence was not investigated by the questionnaire, symptoms of dyspnea improved from 2 to 0, as measured by the modified British Medical Research Council (mMRC) questionnaire [5]). The reticular shadow in the lower field of his chest radiograph and his pulmonary function, including forced vital capacity (FVC), were improved three and six months later, respectively. Because he experienced appetite loss with pirfenidone at a dose of 1800 mg/day, he has been taking 1200–1400 mg/day with a proton pump inhibitor for approximately two years, and has experienced no marked side effects. Since he began pirfenidone treatment, the reticular shadow on his chest and his FVC appeared to have improved, and his condition has been stable for more than two years (Figure 1; Figure 2A). Although levels of fibrotic markers KL-6 and SP-D were temporarily elevated with no symptoms, 14 months after pirfenidone treatment, they normalized under continuous administration of pirfenidone (Figure 2B). Interestingly, his serum periostin levels were not high at the time of his initial admission to our hospital and, in contrast with the decrease in levels of fibrotic markers, were not affected by pirfenidone treatment (Figure 2C,D).

### Consent for Publication

Written informed consent was obtained from the patient for publication of this article and accompanying images. This work was supervised by the Ethics Committee of Gunma University Hospital (No. 13-66) approved on 15 April 2014.

## 3. Discussion

While FIPF is defined as a form of IPF that affects two or more members of the same primary biological family, it is often assumed to have a wider familial definition, with two or more affected relatives [7]. There are two case reports that have described IPF related to Hermansky–Pudlak syndrome treated with pirfenidone, and the effects of pirfenidone varied [8,9]. Boing et al. reported that pirfenidone administration to a patient with FIPF resulted in stable pulmonary function for six months [10].

Currently, fibrotic markers such as KL-6, SP-A, and SP-D have not been utilized as markers for IPF diagnosis and/or follow-up of IPF disease activity. It remains unknown if the decreased levels of fibrotic markers reflect the reduced activity of IPF in response to antifibrotic agents because the relationship between serial analysis of fibrotic markers and the physiological variables of IPF has not yet been established. Previously, Collard et al. reported that changes in clinical and physiological variables, such as symptom-based dyspnea score, predicted FVC percentage, and AaPo2 (alveolar–arterial oxygen gradient), are predictors of overall survival in IPF patients. Furthermore, serial changes in serum KL-6 levels are associated with changes in physiological variables and can predict survival in IPF cases [11]. Recently, Wakamatsu et al. reported that a progressive increase in KL-6 levels correlates with poor survival and a rapid decline of FVC [12]. Sokai et al. reported that the decline of pulmonary function is significantly correlated with the six-month increase in KL-6 values, but not SP-D values, and the progressive increase in KL-6 values in six months is a stronger predictive biomarker of mortality than the baseline KL-6 value [13]. IPF is considered to be a chronic progressive disease, even under treatment with antifibrotic agents. Recently, it has been reported that nintedanib, but not pirfenidone, presented with remarkable improvement of the FVC together with decreased KL-6 value in a patient with IPF [14].

Periostin is known to be involved in the progression and development of IPF [15], and the high levels of serum periostin are significantly associated with the progression of IPF [6,16]. Baseline serum periostin levels are a possible predictive biomarker of the development of honeycombing, although no correlation was found during the six months between progressive changes of serum periostin levels and that of KL-6 or LDH levels in the analysis of 12 patients with IPF [17]. As Okamoto et al. reported [18], serum periostin levels are elevated in the usual interstitial pattern of IP compared with other IP patterns; accordingly, we monitored the patient’s serum periostin, KL-6, and SP-D levels. While antiperiostin antibodies 18A and 17B recognize both inflammatory and fibrotic total periostin, the 20A and 19D antibodies recognize monomeric fibrotic periostin and predict the short-term progression of IPF [6].

It is known that periostin is primarily produced by myofibroblasts, but not by bronchial epithelial cells, in pulmonary fibrosis [19], whereas both KL-6 and SP-D are produced by regenerating type II alveolar cells [20]. Injured type II alveolar epithelial cells release various cytokines that are the key mediators of fibrotic tissue remodeling. The result is enhanced production of extracellular-matrix proteins by myofibroblasts, leading to further remodeling [21]. This difference between periostin and KL-6/SP-D in the producing cells and the mechanism of release may explain the different effects of pirfenidone-induced improvement. Recently, we reported an autopsy case of acute exacerbation of FIPF. Immunohistochemical analysis showed an accumulation of periostin in the severe fibrotic lesion of lungs at autopsy, while serum periostin levels were not elevated on admission, indicating that periostin may be accumulated in progressive fibrotic lesions [22]. The different time course of fibrotic markers after pirfenidone administration, in this case, suggests the possibility that pirfenidone influences the inflammatory or fibrotic response in alveolar epithelial cells. However, further studies are required to clarify the therapeutic mechanisms of antifibrotic agents.

## 4. Conclusions

We reported a case of FIPF treated with pirfenidone, which may contribute to a therapeutic strategy for its management. Antifibrotic agents such as pirfenidone may be useful for the management of FIPF, as well as sporadic IPF. Furthermore, differential changes in the parameters of fibrotic markers KL-6, SP-D, and periostin may suggest a possible mechanism of the antifibrotic effects of pirfenidone for IPF.
